# SmiA is a hybrid priming/scaffolding adaptor for the LonA protease in *Bacillus subtilis*

**DOI:** 10.1016/j.jbc.2022.102045

**Published:** 2022-05-18

**Authors:** Stephen G. Olney, Peter Chien, Daniel B. Kearns

**Affiliations:** 1 Department of Biology, Indiana University, Bloomington, Indiana, USA; 2 Department of Biochemistry and Molecular Biology, University of Massachusetts Amherst, Amherst, Massachusetts, USA

**Keywords:** proteolysis, adaptor protein, ATP-dependent protease, cell motility, BLI, biolayer interferometry, CV, column volume, gDNA, genomic DNA, GST, glutathione-*S*-transferase, Ni, nickel, NTA, nitrilotriacetic acid, TB, Terrific broth, TEV, tobacco etch virus, TKM, Tris, KCl, and MgCl_2_

## Abstract

Regulatory proteolysis targets properly folded clients *via* a combination of *cis*-encoded degron sequences and trans-expressed specificity factors called adaptors. SmiA of *Bacillus subtilis* was identified as the first adaptor protein for the Lon family of proteases, but the mechanism of SmiA-dependent proteolysis is unknown. Here, we develop a fluorescence-based assay to measure the kinetics of SmiA-dependent degradation of its client SwrA and show that SmiA–SwrA interaction and the SwrA degron were both necessary, but not sufficient, for proteolysis. Consistent with a scaffolding adaptor mechanism, we found that stoichiometric excess of SmiA caused substrate-independent inhibition of LonA-dependent turnover. Furthermore, SmiA was strictly required even when SwrA levels were high suggesting that a local increase in substrate concentration mediated by the scaffold was not sufficient for proteolysis. Moreover, SmiA function could not be substituted by thermal denaturation of the substrate, consistent with a priming adaptor mechanism. Taken together, we conclude that SmiA functions *via* a mechanism that is a hybrid between scaffolding and priming models.

Maintaining a properly functioning proteome is essential to cellular fitness, and a variety of proteases degrade proteins in the bacterial cytoplasm. The majority of cytoplasmic proteases are composite enzymes belonging to the Clp/Hsp100 family that consist of an ATP-dependent module responsible for recognizing, unfolding, and translocating a target protein to the generalized peptidase module, which catalyzes peptide bond hydrolysis by a conserved serine/lysine active-site residue (1, 2, 3). The other major family of proteases is the Lon family, which has similar domain architecture as the Clp/Hsp100 family, but the unfoldase and proteolytic domains are fused into a single polypeptide (4, 5, 6, 7). Since proteolysis is irreversible, both families of proteases are subject to specificity determinants that restrict proteolysis to a particular subset of the proteome (8). In general, both families are responsible for the degradation of misfolded proteins using more general recognition features as well as regulatory proteolysis, which invokes target-specific information to direct proteolysis even when properly folded (9).

Misfolded proteins are thought to be discriminated by the presence of exposed hydrophobic residues that would ordinarily be concealed in the protein core (10). Moreover, misfolded protein targets may encode a degron, a short stretch of amino acids typically found at either the N or C terminus of the protein that is specifically bound to and directs degradation by a specific protease (11, 12). Properly folded proteins may also be selectively destroyed as a regulatory mechanism, and such proteins may also encode degrons for protease targeting (13, 14, 15). Degron sequences, while necessary, may be insufficient for high-frequency recognition and turnover of low abundance targets during regulatory proteolysis. In these cases, additional specificity factors called adaptors, defined as one protein that specifically activates the turnover of another protein, may be required (16).

Adaptors typically increase proteolysis of their target protein by a priming mechanism or a scaffolding mechanism (17). Priming adaptors stimulate proteolysis by interaction with either the client or the protease. Those adaptors that bind to the client alter client conformation to create a protease-sensitized state for protease recognition, whereas those that bind to the protease alter protease conformation such that the affinity for a particular client is increased (12). Some protease-priming adaptors bind to and allosterically activate generalized protease activity, thereby increasing the turnover rate of all clients including the regulatory target in question. By contrast, scaffolding adaptors interact with both the client and the cognate protease simultaneously forming a tether to increase the local substrate concentration and enhance delivery, translocation, and eventual destruction of the target (17, 18, 19, 20). Scaffolding adaptor proteins have primarily been characterized for the Clp family of proteases, whereas allosteric enhancers have been primarily reported for the Lon family (15, 21, 22, 23, 24, 25, 26, 27). Recently, the first substrate-specific adaptor protein for the Lon family was reported when the protein SmiA was shown to be obligately required for the proteolysis of SwrA, the master activator of flagellar gene expression in *Bacillus subtilis* (28, 29).

SwrA is a small, basic, and narrowly conserved protein of poorly understood function that activates the promoter of a large operon of genes dedicated to flagellar biosynthesis (30, 31, 32). SwrA requires its partner DNA-binding response regulator DegU, and SwrA-dependent activation occurs over a narrow fourfold dynamic range (31, 33, 34). While the transcriptional effects are subtle, the biological consequences are substantial as SwrA controls the frequency of motile cells in a subpopulation, the density of flagella synthesized per cell, and the ability of a population to migrate over solid surfaces (*i.e.*, swarming motility) (30, 31, 35, 36, 37, 38, 39). The levels of SwrA dictate the degree of flagellar gene activation and are controlled proteolytically in response to environmental input (28, 31, 38). In liquid environments, SmiA-dependent LonA proteolysis restricts SwrA accumulation, and while motile bacteria are produced, the average flagellar number per cell is insufficient to support surface motility. Upon surface contact, proteolytic turnover is relieved, resulting in the accumulation of SwrA that leads to the increase of flagellar gene expression necessary to potentiate swarming. How SwrA proteolysis is activated and/or inhibited is unknown, but we hypothesize that regulation depends on the mechanism by which SmiA functions as an adaptor.

Here, we develop an *in vitro* fluorescence-based turnover assay to show that SmiA exhibits features of both scaffolding and priming mechanisms. We show that SmiA binds to SwrA and promotes optimal turnover when the proteins are present in a roughly 2:1 ratio. At higher concentrations, SmiA inhibited proteolysis, and the inhibition was generalized as LonA activity was diminished even when SwrA was absent. Thus, like a scaffolding adaptor, SmiA bound to both the client and the protease. Simultaneous binding however seemed unrelated to a local increase in protein concentration as even high levels of SwrA were immune to proteolysis when SmiA was absent. SwrA encodes a C-terminal degron sequence that while necessary was not sufficient for turnover, and thermal denaturation of SwrA not only failed to expose the degron but also actually made SwrA resistant to proteolysis even in the presence of SmiA. We conclude that SmiA exhibits features of both mechanistic classes in that it binds to both partners and in so doing, alters each to facilitate SwrA turnover.

## Results

### A fluorescence-based turnover assay for SwrA proteolysis

**Figure 1:**
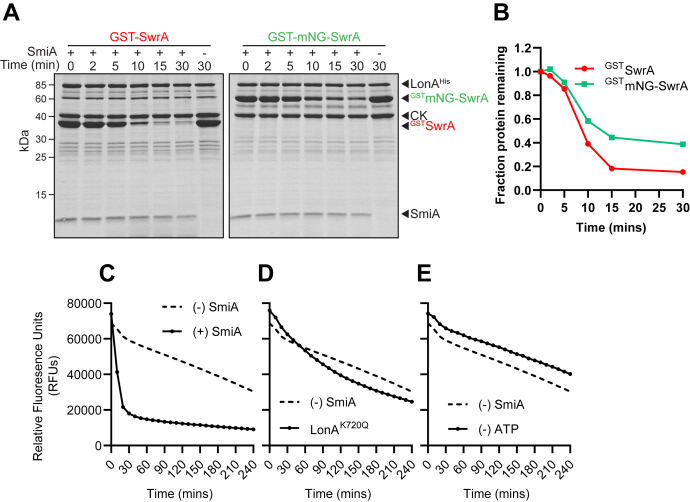
**GST-mNG-SwrA is efficiently degraded in the presence of SmiA and LonA.***A*, Coomassie-stained SDS-PAGE gels of *in vitro* proteolysis reactions of GST-SwrA (*left*) and GST-mNG-SwrA (*right*). The presence or the absence of SmiA in the reaction is indicted by a + or – symbol, respectively, above the lane. Each protein in the reaction is indicated by an annotated caret at the *right*: LonA^His^, creatine kinase (CK), GST-SwrA (^GST^SwrA), GST-mNG-SwrA (^GST^mNG-SwrA), and SmiA. *B*, densitometry analysis of band intensity for either ^GST^SwrA (*black*) or ^GST^mNG-SwrA (*green*) from the corresponding panels for *A*. Fraction of remaining protein was calculated relative to band intensity at time point 0. *C*, *in vitro* proteolysis of GST-mNG-SwrA detected by fluorescence emission in a plate reader. *Lines* indicate fluorescence loss in the presence (*solid line*) or the absence (*dotted line*) of SmiA. *D*, *in vitro* proteolysis of GST-mNG-SwrA detected by fluorescence emission in a plate reader. *Solid line* indicates fluorescence loss in the presence of an active-site mutant of LonA (K720Q), and *dotted line* indicates fluorescence loss in the absence of SmiA. *E*, *in vitro* proteolysis of GST-mNG-SwrA detected by fluorescence emission in a plate reader. *Solid line* indicates fluorescence loss in the presence of a wildtype LonA in the absence of ATP, and *dotted line* indicates fluorescence loss in the absence of SmiA. *Y*-axis is the same for *C*–*E*. GST, glutathione-*S*-transferase.

To determine the mechanism by which SmiA potentiates LonA-dependent proteolysis of SwrA, we first set out to develop a high-throughput assay for *in vitro* proteolysis. Specifically, a fusion protein was made in which the gene encoding mNeonGreen was inserted between the gene encoding the N-terminal glutathione-*S*-transferase (GST) purification epitope and the *swrA* ORF (GST-mNG-SwrA). When expressed, the solution containing the recombinant purified protein was an intense green color suggesting that the fluorophore was functional. To determine whether the GST-mNG-SwrA fusion was susceptible to LonA-dependent proteolysis, an *in vitro* proteolysis assay was conducted, resolved by SDS-PAGE, and band intensity was measured by densitometry. Both GST-SwrA and GST-mNG-SwrA were degraded within 30 min under standard conditions (Fig. 1*A*). Densitometry suggested that GST-mNG-SwrA appeared to be degraded at a slightly slower rate, but importantly, neither protein was degraded in the absence of SmiA (Fig. 1, *A* and *B*). We conclude that the GST-mNG-SwrA fusion was proficient for SmiA-dependent proteolysis and, in an *in vitro* protein turnover assay, behaved similarly to the nonfluorescent standard.

One advantage of the fluorescent fusion is that proteolytic turnover might be quantitatively measured by loss of fluorescence. To determine whether fluorescence level correlated with protein abundance, the *in vitro* proteolysis assay was conducted in a microtiter plate reader. In the absence of SmiA, fluorescence of GST-mNG-SwrA decreased at a low but constant rate, and addition of SmiA caused a dramatic increase in the rate of fluorescence loss (Fig. 1*C*). The SmiA-dependent loss of fluorescence was also LonA dependent as the rate was reduced to near background levels when LonA protein defective in a proteolytic active-site residue (LonA^K720Q^) was used (Fig. 1*D*). Finally, omission of ATP needed for LonA activity resulted in a baseline rate of fluorescence loss comparable to that observed when SmiA was absent (Fig. 1*E*). We infer that the LonA active-site mutant is partially active for promoting fluorescence loss, perhaps because of persistent unfoldase activity, and that fluorescence loss in the absence of SmiA is likely because of photobleaching of the fluorophore. We conclude that loss of GST-mNG-SwrA fluorescence is a suitable proxy for SwrA proteolytic turnover and demonstrates the same basic requirements as have been previously established.

**Figure 2:**
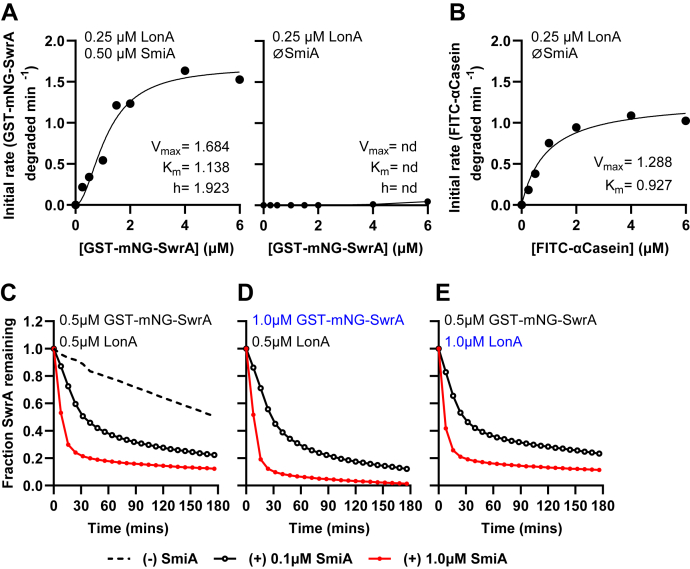
**The *K***_***m***_**for LonA degradation of SmiA–SwrA and α-casein is similar.** Michaelis–Menten kinetic curves for GST-mNG-SwrA in the presence of SmiA (*A*) and FITC-α-casein (*B*). *V*_max_ and *K*_*m*_ are indicated for each reaction within their corresponding panel. The Hill coefficient (h) is also provided in the case of GST-mNG-SwrA degradation. Each data point represents the average rate of *in vitro* turnover assays performed in triplicate. *C*, the fraction of 0.5 μM GST-mNG-SwrA remaining overtime in the absence (*dashed line*) or the presence of 0.1 μM SmiA (*black*, *open circles*) and 1 μM SmiA (*red*, *closed circles*). *D*, the fraction of 1 μM GST-mNG-SwrA remaining overtime. *E*, the fraction of 0.5 μM GST-mNG-SwrA remaining in the presence of 1 μM LonA. Each data point represents the average amount of GST-mNG-SwrA remaining in *in vitro* turnover assays performed in triplicate. GST, glutathione-*S*-transferase.

Another advantage of a fluorescent substrate is that steady-state kinetic parameters can be measured in real time over a range of substrate concentrations. For the *in vitro* reactions, a variable amount of GST-mNG-SwrA was treated with constant 0.25 μM LonA and 0.5 μM SmiA. The arbitrary fluorescence units were converted to molecules of SwrA by use of a standard curve (Fig. S1*A*), and for each substrate concentration, rates were calculated as the slope of the linear part of the degradation curve subtracted by the constant rate attributed to photobleaching. The rate of proteolysis increased with increasing amounts of GST-mNG-SwrA and leveled out at high concentrations consistent with enzyme saturation in Michaelis–Menten kinetics (Fig. 2*A*, *left*). We note that low concentrations of substrate gave rise to a sigmoidal response curve with a Hill coefficient of 1.92 indicative of cooperativity in the system. Nonlinear regression of the resulting curve provided a *V*_max_ value from which the *K*_*m*_ of the reaction was calculated to be 1.14 μM GST-mNG-SwrA. No proteolysis of GST-mNG-SwrA was detected when SmiA was omitted from the reaction (Fig. 2*A*, *right*). For comparison, LonA also nonspecifically degrades misfolded proteins, and *in vitro* proteolysis was performed in parallel with the misfolded protein standard: fluorescently labeled α-casein. The resulting hyperbolic curve was consistent with standard Michaelis–Menten kinetics with a predicted *K*_*m*_ of 0.9 μM α-casein and with previously reported results (40) (Fig. 2*B*). We conclude LonA has similar affinity for SwrA as it does for misfolded protein substrates but only when SmiA is present.

Adaptor proteins are catalytic by iteratively binding to either the substrate, protease, or both proteins to potentiate proteolysis. To determine whether SmiA acts as a single-use co-substrate or is capable of catalyzing multiple rounds of proteolysis, 0.5 μM GST-mNG-SwrA was subjected to proteolysis by either excess (1 μM) or substoichiometric amounts (0.1 μM) of SmiA. Both conditions resulted in comparable levels of GST-mNG-SwrA remaining after completion of the reaction, albeit SwrA proteolysis occurred at different rates (Fig. 2*C*). Moreover, the residual GST-mNG-SwrA remaining in the reaction at the two different SmiA concentrations was largely unchanged by increasing either the amount of substrate (Fig. 2*D*) or the amount of protease (Fig. 2*E*). Thus, the amount of residual GST-mNG-SwrA remaining after the reaction was not proportional to the amount of SmiA added to the reaction, regardless of the reaction conditions. Finally, SmiA levels did not appear to decrease over the course of the *in vitro* proteolysis reaction (Fig. 1*A*). We conclude that the total amount of SwrA degraded was not dependent on the amount of SmiA, and that SmiA is recycled to catalyze multiple rounds of LonA-dependent SwrA proteolysis.

### SmiA interacts with LonA transiently

**Figure 3:**
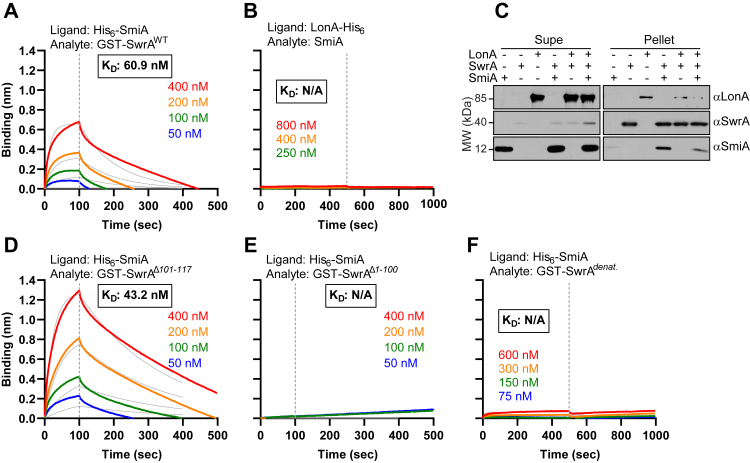
**SmiA and SwrA interact in protein–protein interaction assays.***A*, biolayer interferometry (BLI) association/dissociation curves in which His_6_-SmiA was coupled to an HIS1K biosensor and monitored over time in the presence of increasing concentration of GST-SwrA^WT^. *B*, BLI association/dissociation curves in which LonA-His_6_ was coupled to an HIS1K biosensor and monitored over time in the presence of increasing concentration of SmiA. *C*, protein pull-down assay detected by Western blot analysis using αLonA, αSwrA, and αSmiA primary antibodies. Each lane indicates the presence (+) or absence (−) of purified LonA^K720Q^-His_6_, GST-SwrA, and SmiA proteins mixed for 30 min and then presented to GST-reactive Glutathione-Sepharose resin. Supernatants were saved, and pellets were separately eluted with 20 mM glutathione. *D*, BLI association/dissociation curves in which His_6_-SmiA was coupled to an HIS1K biosensor and monitored over time in the presence of increasing concentration of GST-SwrA^Δ101–117^. *E*, BLI association/dissociation curves in which His_6_-SmiA was coupled to an HIS1K biosensor and monitored over time in the presence of increasing concentration of GST-SwrA^Δ1–100^. *F*, BLI association/dissociation curves in which His_6_-SmiA was coupled to an HIS1K biosensor and monitored over time in the presence of increasing concentration of thermally denatured GST-SwrA (GST-SwrA^*denat*^). For all BLI data, the *dashed gray line* indicates the time point when the biosensor was introduced to buffer lacking analyte (dissociation step). GST, glutathione-*S*-transferase.

SmiA might function catalytically as either a scaffolding adaptor or priming adaptor. Consistent with either model, SwrA was shown to interact with His-SmiA immobilized on a biolayer interferometry (BLI) biosensor with high affinity, and the best fit curves were obtained using a 1:1 binding model (Fig. 3*A*). To explore the possibility of a scaffolding mechanism, we sought to determine whether SmiA and LonA directly interact. When LonA-His was mounted on a BLI biosensor, addition of ATP and SmiA did not indicate a positive interaction (Fig. 3*B*). Since BLI relies on changes in the mass of the complex and SmiA is much smaller than hexameric LonA, the negative result may have been because of the small size differential upon SmiA addition. In a parallel approach, we attempted a protein pull-down assay in which GST-SwrA was loaded on a Glutathione-Sepharose matrix, and combinations of SmiA and the active-site mutant version of LonA^K720Q^ were added. Whereas SmiA was retained in the pellet when GST-SwrA was present, LonA^K720Q^ was not retained above the background levels (Fig. 3*C*). While SmiA and SwrA interact, interaction between SmiA and LonA remains undetermined with affinity-based approaches. We conclude that either SmiA and LonA do not directly interact or the interaction is too transient to capture by the methods used.

**Figure 4:**
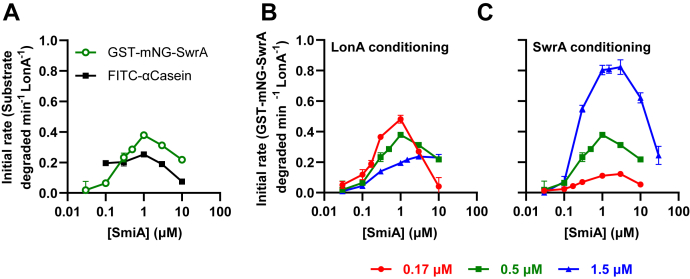
**SmiA stimulates SwrA proteolysis but becomes generally inhibitory at high concentration.***A*, the rate of 0.5 μM GST-mNG-SwrA (*green*) and 0.25 μM FITC-α-casein (*black*) proteolysis as a function of SmiA concentration in the presence of 0.5 μM LonA hexamers. *B*, the rate of 0.5 μM GST-mNG-SwrA degradation as a function of SmiA concentration in the presence of 0.17 μM (*red*), 0.5 μM (*green*), and 1.5 μM (*blue*) LonA hexamers. *C*, the rate of 0.17 μM (*red*), 0.5 μM (*green*), and 1.5 μM (*blue*) of GST-mNG-SwrA degradation as a function of SmiA concentration in the presence 0.5 μM LonA hexamers. Relative fluorescence was converted to molecules of fluorescent substrate using a standard curve (Fig. S1), and initial velocity was derived from the maximum slope of each degradation curve. Each data point represents the average rate of *in vitro* turnover assays performed in triplicate, and error bars represent SD. GST, glutathione-*S*-transferase.

As an alternative to direct observation by protein–protein interaction assay, we attempted to use kinetic analysis to infer protein interaction. To do so, a constant 0.5 μM GST-mNG-SwrA was treated with constant 0.5 μM LonA and a variable amount of SmiA in the reaction. The rate of SwrA proteolysis increased with increasing amounts of SmiA until the two proteins were at roughly a 2:1 SmiA:SwrA ratio, at which point addition of greater amounts of SmiA to the reaction reduced the overall rate of proteolysis (Fig. 4*A*). To determine whether the SmiA concentration-dependent effect on proteolysis was specific for SwrA, a similar curve was generated using a constant amount of fluorescent α-casein in the reaction. Unlike that observed with GST-mNG-SwrA, addition of low levels of SmiA did not substantially increase the rate of fluorescent α-casein hydrolysis (Fig. 4*A*). High levels of SmiA in the reaction, however, inhibited the proteolysis of fluorescent FITC-α-casein (Fig. 4*A*). We conclude that SmiA specifically accelerates proteolysis for its cognate but becomes generally inhibitory at high concentrations. We further conclude that SmiA and LonA are capable of at least transient interaction, as SmiA inhibited LonA activity even when SwrA was absent likely by occlusion of the LonA-substrate recognition site(s).

If SmiA interacts with LonA, SmiA proteolytic inhibition might be relieved by titrating LonA. To determine whether SmiA inhibition was governed by LonA levels, *in vitro* proteolysis experiments were performed with constant SwrA (0.5 μM), variable SmiA, and either a threefold increase (1.5 μM) or a threefold decrease (0.17 μM) in LonA concentration. Low levels of LonA were the most responsive to SmiA titration, both in terms of substoichiometric activation and superstoichiometric inhibition. As LonA levels increased, both the activating and inhibitory roles of SmiA were diminished (Fig. 4*B*). A similar experiment was performed altering the ratio of SwrA to SmiA in the reaction, in which a constant 0.5 μM LonA, variable SmiA, and either a threefold increase (1.5 μM) or threefold decrease (0.17 μM) in GST-mNG-SwrA levels were provided. Turnover rate increased proportionally with both SmiA and GST-mNG-SwrA concentrations, and for each reaction series, peak turnover rate was achieved when the two were at or near equimolar ratio (Fig. 4*C*). We conclude that the activity of SmiA, both in terms of activation and inhibition, is primarily dependent on the amount of LonA present in the system. We conclude that kinetic evidence supports an interaction between SmiA and LonA, and we infer that this interaction was not observed by direct assays perhaps because contact is transient.

**Figure 5:**
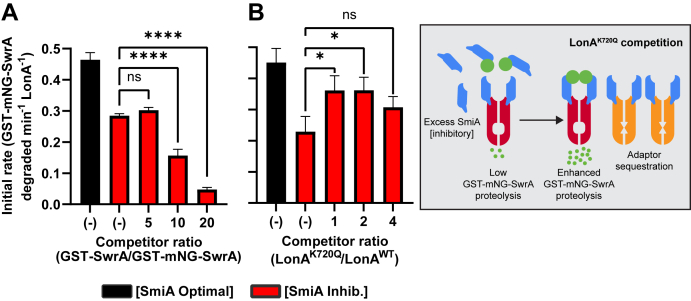
**GST-mNeonGreen SwrA proteolysis can be competitively inhibited either by the addition of unlabeled substrate or an active-site mutant of LonA.***A*, rate of 0.5 μM GST-mNG-SwrA proteolysis in the presence 0.5 μM SmiA and 0.5 μM LonA and competitor GST-SwrA expressed as a concentration ratio relative to the fluorescent substrate. *B*, *left*, rate of 0.5 μM GST-mNG-SwrA proteolysis in the presence of 0.5 μM SmiA and 0.5 μM LonA plus competitor LonAK720Q-His levels expressed as a ratio relative to functional enzyme. *B*, *right*, excess SmiA (*blue*) inhibits LonA (*red*) degradation of GST-mNG-SwrA (*green*). Addition of inactive LonA^K720Q^ (*orange*) sequesters excess SmiA and restores GST-mNG-SwrA proteolysis. Each data point represents the average rate of *in vitro* turnover assays performed in triplicate, and error bars represent the SD. Statistical analysis: ∗ indicates a difference significance of *p* = 0.0014, ∗∗∗∗ indicates a difference significance of *p* < 0.0001; ns indicates that the difference is not significant. GST, glutathione-*S*-transferase.

To investigate the possibility of transient SmiA–LonA interaction, we performed *in vitro* proteolysis of GST-mNG-SwrA in the presence of inhibitory levels of SmiA and titrating in candidate competitive substrate and protease inhibitors. Increasing amounts of nonfluorescent GST-SwrA gradually inhibited the turnover of the fluorescent protein suggesting that both versions functioned as substrates and that the nonfluorescent form titrated the pool of both the SmiA adaptor and the LonA protease (Fig. 5*A*). Addition of low levels of the proteolytically inactive mutant LonA^K720Q^ however actually increased the proteolytic rate suggesting that the protease-defective subunits titrated the SmiA inhibitory effect on the active subunits in the reaction (Fig. 5*B*). Higher levels of added LonA^K720Q^ appeared to lessen the antagonism perhaps because subunit exchange, if any, between proteolytic defective and wildtype LonA complexes began to inhibit the overall rate (Fig. 5*B*, *left*). We conclude that inactive LonA^K720Q^ sequesters and effectively dilutes the amount of SmiA that would otherwise inhibit the rate of proteolysis (Fig. 5*B*, *right*). We further conclude that the effect is transient as while LonA^K720Q^ titrates SmiA it also allows for the recycling of the SmiA pool and a net overall increase in SwrA turnover.

### SmiA–SwrA interaction is necessary for degron presentation

**Figure 6:**
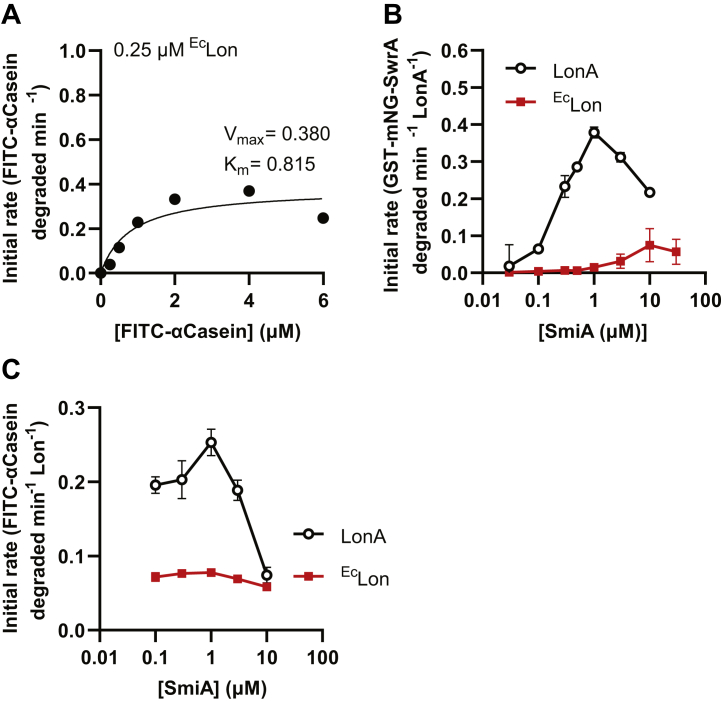
**SmiA stimulates GST-mNG-SwrA turnover by *Escherichia coli* Lon when present at high concentration.***A*, Michaelis–Menten kinetic curves for FITC-α-casein degraded by *E. coli* Lon protein. *V*_max_ and *K*_*m*_ are indicated for each reaction within the panel. *B*, the rate of 0.5 μM GST-mNG-SwrA and *C*, 10 μM FITC-α-casein proteolyzed by either 0.5 μM *Bacillus subtilis* LonA (*black*) or 0.5 μM *E. coli* Lon (*red*) as a function of SmiA concentration. Each data point represents the average rate of *in vitro* turnover assays performed in triplicate, and error bars represent SD. GST, glutathione-*S*-transferase.

SmiA-dependent proteolysis was previously shown to require *B. subtilis* LonA, perhaps consistent with specific interactions between the two proteins (28). The Lon family of proteins however is highly conserved in bacteria, and thus, we infer that specificity, if present, must be relegated to areas of low conservation (Fig. S2). To re-explore SmiA specificity using our quantitative fluorescence-based assay, GST-mNG-SwrA was presented to purified *Escherichia coli* ^Ec^Lon protease. The ^Ec^Lon prep was proteolytically active as it degraded FITC-α-casein with a *K*_*m*_ similar to that observed for LonA (Fig. 6*A*). Consistent with previous observations, ^Ec^Lon was unable to proteolyze GST-mNG-SwrA in the presence of a stoichiometric amount of SmiA in the reaction (Fig. 6*B*). GST-mNG-SwrA proteolysis could be observed however, albeit at suboptimal rates, when the concentration of SmiA was so high that it would ordinarily inhibit the activity of LonA from *B. subtilis* (Fig. 6*B*). We conclude that SmiA can in fact potentiate proteolysis of SwrA by *E. coli* Lon, but that the affinity between the protease and the adaptor was greatly reduced relative to the biological cognate. In addition, the inhibitory effect of superstoichiometric amounts of SmiA on *B. subtilis* LonA was not observed for ^Ec^Lon (Fig. 6*C*) when FITC-α-casein was used as a substrate. We further conclude that excess SmiA inhibits proteolysis of *B. subtilis* Lon by interfering with the protease in a manner that depends on the interaction affinity.

**Figure 7:**
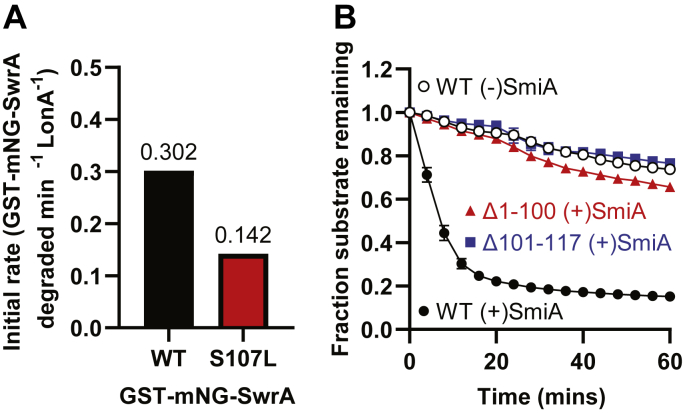
**The SwrA C-terminal degron is necessary but not sufficient for LonA-dependent proteolysis.***A*, the initial rate of proteolytic turnover for 0.5 μM GST-mNG-SwrA (*black*) and GST-mNG-SwrA^S107L^ (*red*). Each data point represents the average rate of *in vitro* turnover assays performed in triplicate. *B*, fluorescent protein degradation of GST-mNG-SwrA^Δ1–100^ and SwrA^Δ101–117^. Each data point represents the average rate of *in vitro* turnover assays performed in triplicate, and error bars represent SD. Note some error bars are so small they do not leave the boundaries of the symbol. GST, glutathione-*S*-transferase.

Interaction affinity with a protease is governed by extrinsic factors like adaptors and intrinsic properties of the target such as a degron. SwrA is a 117 amino acid protein, and previous genetic analysis of residues necessary for SmiA-dependent LonA proteolysis was localized to the last 17 amino acids inferred to constitute a degron (29). To re-explore the degron hypothesis using our quantitative fluorescence-based assay, a mutant in the C-terminal region of SwrA previously shown to resist proteolysis both *in vivo* and *in vitro* (SwrA^S107L^) was fused to GST-mNeonGreen and purified. Quantitative analysis indicated that GST-mNG-SwrA^S107L^ was not fully resistant to proteolysis but reduced the rate twofold relative to wildtype (Fig. 7*A*). We infer that the twofold reduction in rate was sufficient to permit SwrA^S107L^ accumulation *in vivo*, but the reduced rate of proteolysis was previously difficult to detect because of the large amounts of substrate protein necessary *in vitro* when SDS-PAGE and Coomassie staining was used as a reporter (29). We conclude that at least one previously isolated mutant in the degron region reduced but did not abolish proteolysis of SwrA.

To further explore the hypothesis that the C terminus of SwrA functioned as a degron, the last 17 amino acids of SwrA (SwrA^Δ101–117^) were deleted from the GST-mNG construct, and the GST-mNG-SwrA^Δ101–117^ protein was purified. GST-mNG-SwrA^Δ101–117^ failed to be degraded *in vitro* either in the presence or in the absence of SmiA (Fig. 7*B*, *blue*). While proteolysis was abrogated, SmiA and GST-SwrA^Δ101–117^ retained a positive protein–protein interaction by BLI analysis (Fig. 3*D*). We conclude that the sequence contained within the last 17 amino acids of SwrA is essential for proteolysis but not interaction with the adaptor. Previous work indicated that the degron region of SwrA was not sufficient to promote proteolysis when appended to an artificial substrate like GST (GST-SwrA^Δ1–100^) (29). To re-explore sufficiency using our quantitative fluorescence-based assay, we used a GST-mNG-SwrA^Δ1–100^ variant protein that appended only the degron region of SwrA to GST-mNG. As previously reported, addition of the last 17 amino acids of SwrA to GST-mNeonGreen did not stimulate loss of fluorescence in *in vitro* proteolysis (Fig. 7*B*, *red*). Finally, no interaction was detected between His-SmiA and GST-SwrA^Δ1–100^ (Fig. 3*E*). We conclude that while the C terminus of SwrA behaves as a degron, it is necessary but not sufficient for LonA recognition and/or proteolysis. Moreover, SmiA interacts with SwrA outside the C terminus, and we infer that this interaction is necessary for proper presentation of the degron sequence.

**Figure 8:**
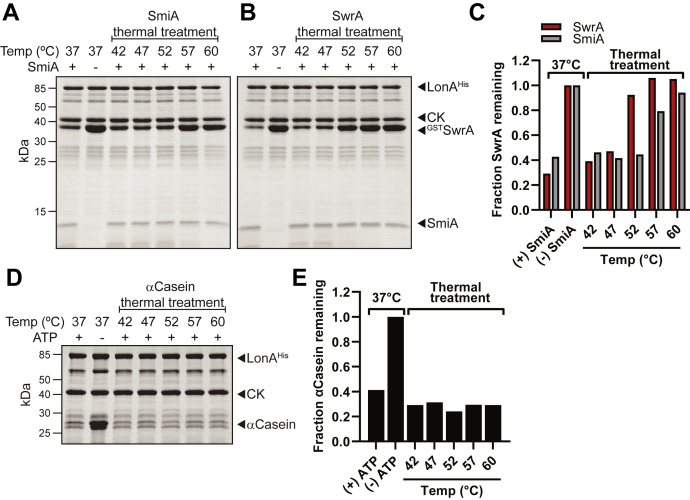
**Thermal denaturation of either SmiA or SwrA inhibits SwrA proteolysis.***A*, *in vitro* proteolysis reactions in which 3 μM SwrA was incubated for 15 min at the indicated temperature prior to addition of 1 μM LonA and the presence (+) or absence (−) of 1 μM SmiA. *B*, *in vitro* proteolysis reactions in which 1 μM SmiA was incubated for 10 min at the indicated temperature prior to addition of 3 μM SwrA and 1 μM LonA. *C*, densitometry analysis of GST-SwrA remaining relative to the control lacking SmiA for data from *A* (*red*) and *B* (*gray*). *D*, *in vitro* proteolysis reactions in which 10 μM a-casein was incubated for 15 min at the indicated temperature prior to addition of 1 μM LonA and the presence (+) or absence (−) of ATP. *E*, densitometry analysis of a-casein remaining relative to the control lacking ATP for data from *D*. GST, glutathione-*S*-transferase.

To determine whether the requirement of SmiA could be bypassed, thermal denaturation was attempted as an alternative means of degron presentation. To test this hypothesis, GST-SwrA was incubated at a variety of temperatures above the standard condition of 37 °C for 15 min before being added to a standard LonA *in vitro* proteolysis assay. Each sample was resolved by SDS-PAGE, and the amount of SwrA protein remaining was detected by Coomassie staining. Incubation of SwrA at 42 and 47 °C exhibited little reduction in proteolysis, but treatment at 52 °C and above resulted in near complete stability (Fig. 8, *B* and *C*). We infer that SwrA conformation is important for proteolytic targeting and that a protease-sensitive state cannot be achieved simply by denaturation. Unlike SwrA, thermal denaturation of the inherently disordered substrate α-casein did not abolish proteolytic turnover (Fig. 8, *D* and *E*), and thus, denaturation of SwrA likely abolished proteolysis by abrogating SmiA interaction. Consistent with an interaction failure, thermal denaturation of GST-SwrA above the threshold temperature also abolished interaction with His-SmiA mounted on a BLI biosensor tip (Fig. 3*F*). Finally, incubation of SmiA at 52 °C and below exhibited little reduction in SwrA proteolysis, but treatment at 57 °C and above resulted in near complete stability of SwrA (Fig. 8, *A* and *C*). We conclude that when SmiA interacts with SwrA, the C-terminal degron is exposed and presented to LonA as a transient three-protein supercomplex necessary for proteolysis.

## Discussion

SmiA of *B. subtilis* is the first substrate-specific adaptor protein reported for the Lon family of proteases, and the mechanism by which SmiA potentiates proteolysis of its client SwrA was unknown (28). Adaptors are well known and characterized for the Clp family of proteases however, and these adaptors tend to fall into two general mechanistic classes: scaffolding and priming adaptors. Scaffolding adaptors bind to both the substrate and protease simultaneously and accelerate proteolysis by increasing local substrate concentration at the enzyme. In contrast, priming adaptors either bind exclusively to the substrate to expose a protease-sensitive degron sequence or bind exclusively to the protease to allosterically alter conformation to expose a degron-recognition pocket or stimulate overall protease activity (17). Here, we show that SmiA optimally potentiates the proteolysis of SwrA when in a 2:1 ratio relative to the cargo. Moreover, we provide kinetic evidence to show that SmiA exhibits properties consistent with both scaffolding and priming adaptors and appears to confer LonA-specific degradation of SwrA as a hybrid of the two mechanisms.

Consistent with a scaffolding adaptor, SmiA interacts with both the client SwrA and the protease LonA. While interaction between SmiA and SwrA can be directly observed, the evidence for interaction with LonA was kinetic and indirect (Fig. 3, *A*–*C*). Specifically, SmiA becomes generally inhibitory when in stoichiometric excess of LonA, and SmiA inhibition was titrated by adding additional inactive LonA complexes to the reaction (Figs. 4*A* and 5*B*). Ultimately, we conclude that the SmiA–LonA interaction is likely transient, as low levels of additional nonfunctional LonA titrated excess SmiA while permitting the recycling of functional complexes and accelerating the net reaction (Fig. 5*B*). It is unclear whether SmiA-excess–dependent inhibition is biologically relevant as SmiA expression appears to be SwrA activated, artificial expression of SmiA confers no phenotype, and phenotypes conferred by the mutation of *B. subtilis* LonA besides motility inhibition are unknown. Regardless, inhibition of client proteolysis in the presence of excess adaptor is a feature observed for those that function by a scaffolding mechanism, as excess occupancy of the adaptor–protease complex restricts cargo delivery (20). The direct detection of scaffolding adaptor–protease interactions however is rare in the literature likely because of similar transient contacts and the dynamic exchange that permits adaptors to function catalytically, potentially under-reporting the prevalence of scaffolds (18, 19, 41). While SmiA interacts with both the client and protease, we infer that SmiA likely does not simply act to increase local SwrA concentration because the SmiA requirement cannot be bypassed, even when *in vitro* SwrA concentrations are artificially high (Fig. 2*A*, *right*).

**Figure 9:**
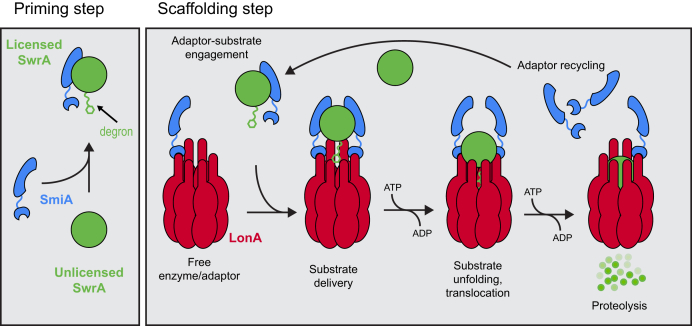
**Mechanism of SmiA-dependent LonA proteolysis of SwrA.** In the absence of SmiA binding, SwrA remains in a protease-resistant state (unlicensed). Either constitutive or concurrent binding of SmiA and LonA to SwrA alters SwrA conformation to reveal a degron (licensed) that is then presented to the LonA in a protease-sensitive state for degradation. SmiA is then released and catalyzes multiple rounds of SwrA proteolysis.

Consistent with a priming adaptor, SmiA binds to the SwrA client to present a C-terminal degron sequence. A C-terminal degron was supported by previously isolated point mutations in the SwrA C terminus (*e.g.*, SwrA^S107L^) that were sufficient to elevate SwrA levels *in vivo*, but here, we show that a representative mutant only reduced *in vitro* turnover twofold (29) (Fig. 7*A*). Nonetheless, we further strengthen the C-terminal degron requirement by demonstrating that deletion of the last 17 amino acids of SwrA (SwrA^Δ101–117^) rendered the protein immune to proteolysis. While the degron is necessary for LonA degradation, it was not sufficient as appendage of the final 17 amino acids to GST-mNeonGreen (GST-mNG-SwrA^Δ1–100^) was insufficient to promote turnover. Finally, thermal denaturation could not artificially expose the degron on native SwrA, and we further demonstrate that the native conformation of both SwrA and SmiA was important for LonA presentation. We therefore conclude that SmiA also acts as a priming adaptor, to either expose the degron on SwrA, put LonA in a conformation able to register the degron, or both. We ultimately arrive at a hybrid model of scaffolding and priming mechanisms whereby SmiA both delivers and conformationally alters its client to license proteolysis (Fig. 9).

Proteolytic adaptor proteins are thought to behave primarily as either priming or scaffolding factors, and thus, the hybrid model presented here may seem unusual when compared with other systems. Only one other specificity factor for the Lon family of proteases, HspQ of *Yersinia pestis*, has been reported and appears quite different from SmiA as it binds to Lon and stimulates the overall rate of proteolysis for a variety of substrates (40). In *B. subtilis*, the adaptor MecA was originally thought to primarily function by binding to its client ComK, but it also binds to and promotes heximerization of the ClpC unfoldase subunit of the ClpCP complex (42, 43, 44, 45). Thus, like SmiA, it may exhibit simultaneous priming and scaffolding activity, but unlike SmiA, MecA stimulates ClpCP-mediated proteolysis of multiple targets, including MecA itself (45, 46). Also in *B. subtilis*, the adaptor YjbH appears to be a priming adaptor as it binds to and licenses the transcription factor Spx for proteolysis by the ClpXP complex (18, 47, 48). We note however that while direct interaction between YjbH–Spx and ClpXP has been difficult to detect, the lack of a positive result in classical protein–protein interaction assays does not necessarily rule out the kind of transient scaffolding interactions described here (49). We suspect that detailed kinetic studies of other adaptors may reveal more hybrid mechanisms.

Regulatory proteolysis invokes control over the degradation of the target protein, and SwrA proteolysis appears to be regulated by environmental factors (28). For example, a basal level of SwrA activates moderate expression of flagellar basal body proteins to support swimming motility in liquid, but SwrA accumulates to a higher level on surfaces to support swarming by further increasing flagellar number. We infer that the transition from liquid to a swarm agar surface somehow reduces proteolytic licensing by SmiA but if, and how, SmiA is regulated is unknown. SmiA could be activated in liquid to keep SwrA at a moderate level, and SmiA seems to be coregulated with flagellar structural proteins creating the potential for homeostatic feedback. How homeostatic restriction would be abrogated on surfaces is unclear, and the loss-of-function genetic screen that identified modulators of SwrA levels only identified SmiA and LonA. We infer that the absence of other candidates argues against a putative activator that would phenocopy SmiA (50). Alternatively, SmiA activity could be inhibited on a solid surface, thereby allowing SwrA to accumulate. Negative regulation is likely as other adaptors have been shown to be inhibited either by a partner switch with small antiadaptor proteins or by covalent post-translational modification of the adaptor (9, 51, 52, 53, 54). Whatever the mechanism of control, SmiA activity seems to be governed by extracytoplasmic information as SmiA inhibits swarming motility when cells are disrupted for particular peptidoglycan hydrolases (50). We presume there exists an as-yet-undiscovered component of LonA regulatory proteolysis that somehow participates in transduction of extracellular information across the cell membrane.

## Experimental procedures

### Strains and growth conditions

*E. coli* strains were grown in lysogeny broth (10 g tryptone, 5 g yeast extract, and 5 g NaCl/ per l) or on lysogeny broth plates fortified with 1.5% Bacto agar (BD Scientific) at 37 °C. When appropriate, antibiotics were added at the following concentrations: 25 μg/ml chloramphenicol, 50 μg/ml carbenicillin, 25 μg/ml kanamycin, or 100 μg/ml ampicillin.

### Strain construction

All PCR products were amplified from *B. subtilis* genomic DNA (gDNA) or *E. coli*-generated plasmid DNA from the indicated strains. All plasmids used in this study are listed in Table S2. All primers used in this study are listed in Table S3.

### Protein expression plasmids

To generate the SmiA expression construct, a PCR fragment containing the *smiA* ORF was amplified from DK1042 gDNA using primers 6497 and 6498. The amplicon was digested with EcoRI and NcoI and ligated into the EcoRI and NcoI sites of the pHis-parallel2 vector to generate pSS920.

To generate the LonA^K720Q^-6XHis expression construct, a PCR fragment with the *lonA* ORF containing the K720Q mutation was amplified from DK3297 gDNA using primers 4574 and 4575. The amplicon was digested with EcoRI and NcoI and ligated into the EcoRI and NcoI sites of the pET28a vector to generate pSO31.

To generate the GST-mNeonGreen-SwrA expression construct, a PCR fragment containing the mNeonGreen ORF and the sequence encoding the Waldo linker (GGATCCGC TGGCTCCGCTGCTGGTTCTGGCGAATTC) was amplified from pDP427 plasmid DNA with primers 7546 and 7547. The amplicon was inserted into the BamHI restriction site of pSM94 *via* Gibson assembly to generate pSO45.

To generate SwrA mutant expression constructs, PCR fragments containing the desired mutations were amplified from the GST-mNeonGreen-SwrA template (pSO45) or the GST-SwrA template (pSM94) with primer pairs 7581/7582 (GST-mNeonGreen-SwrA^S107L^) (GST-SwrA^S107L^), 7625/7626 (GST-mNeonGreen-SwrA^Δ1–100^), and 7627/7628 (GST-mNeonGreen-SwrA^Δ101–117^) (GST-SwrA^Δ101–117^). The linear PCR fragments were DpnI treated and circularized by Gibson assembly to yield pSO46, pSO47, pSO51, pSO52, and pSO53, respectively. The assembled products were subsequently passaged though electrocompetent *E. coli* (DH5α).

### Protein purification

#### SmiA

The His_6_-tobacco etch virus (TEV)-SmiA protein expression vector pSS920 was transformed into Rosetta-gami *E. coli* and grown to an absorbance of ∼0.5 at 600 nm in 1 l of Terrific broth (TB) (900 ml deionized water, 24 g yeast extract, 12 g tryptone, 4 ml glycerol, and 100 ml potassium phosphate solution), induced with 1 mM IPTG, and grown overnight at 16 °C. Cells were pelleted and resuspended in SmiA lysis buffer (25 mM Tris [pH 8.0], 200 mM NaCl, 10 mM imidazole, and 10% glycerol), and PMSF was added to a final concentration of 1 mM. The lysate was subsequently frozen and stored at −80 °C. The frozen cell pellet was thawed and subjected to lysis by emulsification. Lysed cells were clarified by centrifugation at 14,000*g* for 30 min, and the cleared supernatant was combined with equilibrated nickel–nitrilotriacetic acid (Ni–NTA) resin (Novagen). The lysate–resin mixture was added to a 1 cm separation column (Bio-Rad) and allowed to pack. The flow through was collected and subsequently reapplied to the packed resin bed twice. The column was washed with three column volumes (CVs) of SmiA wash buffer (25 mM Tris [pH 8.0], 200 mM NaCl, 20 mM imidazole, and 10% glycerol). Immobilized His_6_-TEV-SmiA was eluted from the resin with SmiA elution buffer (25 mM Tris [pH 8.0], 200 mM NaCl, 200 mM imidazole, and 10% glycerol), and the elution fractions were separated on a 15% SDS-PAGE gel followed by Coomassie staining. The appropriate elution fractions were then pooled and concentrated to ∼2 ml. The concentrate was further purified *via* size-exclusion chromatography on a Superdex 75 16/60 (GE Healthcare) using SmiA storage buffer (20 mM Tris [pH 8.0], 200 mM NaCl, 10% glycerol, and 1 mM DTT). Peak fractions were collected and subjected to cleavage by the TEV protease overnight at 4 °C. The cleavage reaction was applied to ∼2 ml (bed volume) Ni–NTA resin in a 1 cm separation column, and the cleaved SmiA protein was collected *via* subtractive immobilized metal affinity chromatography. The SmiA protein was then buffer exchanged and concentrated using a 3000 Da cutoff Amicon concentrator column with SmiA storage buffer. Aliquots were snap frozen and stored at −80 °C. Protein concentration was determined by Bradford assay (Bio-Rad).

#### GST-SwrA

The GST-SwrA protein expression vector pSM94 was transformed into chemically competent BL21 *E. coli* and grown to an absorbance of ∼0.5 at 600 nm in 1 l of TB, induced with 1 mM IPTG, and grown overnight at 16 °C. Cells were pelleted and resuspended in GST-SwrA lysis buffer (25 mM Tris [pH 8.0], 200 mM NaCl, 1 mM EDTA, and 10% glycerol), and PMSF was added to a final concentration of 1 mM. The lysate was subsequently frozen and stored at −80 °C. The frozen cell pellet was thawed and subjected to lysis by emulsification. Lysed cells were clarified by centrifugation at 14,000*g* for 30 min, and the cleared supernatant was combined with equilibrated Glutathione-Sepharose resin (GE Healthcare) and incubated overnight at 4 °C. The lysate–resin mixture was added to a 1 cm separation column (Bio-Rad) and allowed to pack. The column was washed with 1 CV of GST-SwrA wash buffer (25 mM Tris [pH 8.0], 250 mM NaCl, 1 mM EDTA, 10% glycerol, and 0.1% NP-40) followed by three CVs of GST-SwrA elution buffer (without glutathione) (25 mM Tris [pH 8.5], 250 mM NaCl, 1 mM EDTA, and 10% glycerol). Bound GST-SwrA was eluted from the resin with GST-SwrA elution buffer containing 20 mM glutathione And the elution fractions were separated on a 15% SDS-PAGE gel followed by Coomassie staining. The appropriate elution fractions were then pooled and buffer exchanged into GST-SwrA storage buffer (25 mM Tris [pH 8.0], 250 mM NaCl, 10 mM MgCl_2_, 10% glycerol, and 1 mM DTT) using a HiTrap Desalting column (GE Healthcare). The protein sample was collected and clarified through a 0.22 μm syringe filter and subsequently concentrated using a 3000 Da cutoff Amicon concentrator column. GST-SwrA was snap frozen and stored at −80 °C. Protein concentration was determined by Bradford assay (Bio-Rad).

#### GST-mNG-SwrA variants

The GST-mNG-SwrA protein expression vectors pSO45, pSO46, pSO51, and pSO52 were transformed independently into chemically competent BL21 (+precursor tRNA) *E. coli* and grown to an absorbance of ∼0.5 at 600 nm in 1 l of TB, induced with 1 mM IPTG, and grown overnight at 16 °C. Cells were harvested, lysed, and clarified as described previously. Protein was purified by affinity chromatography and buffer exchanged as described previously.

#### LonA-His_6_

The protein expression vectors pACB60 (LonA-His_6_) or pSO31 (LonA^K720Q^-His_6_) were transformed independently into chemically competent BL21 (+precursor tRNA) *E. coli* and grown to an absorbance of ∼0.5 at 600 nm in 1 l of TB, induced with 1 mM IPTG, and grown overnight at 16 °C. Cells were pelleted and resuspended in LonA lysis buffer (25 mM Tris [pH 8.0], 200 mM NaCl, 100 mM KCl, 10 mM MgCl_2_, 10 mM imidazole, and 10% glycerol), and DTT was added to a final concentration of 1 mM. The lysate was subsequently frozen and stored at −80 °C. The frozen cell pellet was thawed and subjected to lysis by emulsification. Lysed cells were clarified by centrifugation at 14,000*g* for 30 min, and the cleared supernatant was combined with equilibrated Ni–NTA resin. The lysate–resin mixture was added to a 1 cm separation column (Bio-Rad) and allowed to pack. The column was washed with one CV of LonA lysis buffer and three CVs of LonA wash buffer (25 mM Tris [pH 8.0], 200 mM NaCl, 100 mM KCl, 10 mM MgCl_2_, 50 mM imidazole, and 10% glycerol). Immobilized protein was eluted from the resin with LonA elution buffer (25 mM Tris [pH 8.0], 200 mM NaCl, 100 mM KCl, 10 mM MgCl_2_, 500 mM imidazole, and 10% glycerol), and the elution fractions were separated on a 15% SDS-PAGE gel followed by Coomassie staining. The appropriate elution fractions were then pooled and buffer exchanged into LonA storage buffer (50 mM Tris [pH 8.0], 300 mM NaCl, 100 mM KCl, 10 mM MgCl_2_, 10% glycerol, and 1 mM DTT) using a HiTrap desalting column (GE Healthcare). The protein sample was collected and clarified through a 0.45 μm syringe filter and subsequently concentrated to ∼3 ml using a 50,000 Da cutoff Amicon concentrator column. LonA-His_6_ or LonA^K720Q^-His_6_ protein samples were snap frozen and stored at −80 °C. Protein concentration was determined by Bradford assay (Bio-Rad).

### Gel-based *in vitro* proteolysis assay

SwrA proteolytic degradation assays containing GST-SwrA (1 μM), SmiA (1 μM), and LonA_6_ (0.17 μM as a hexamer) were assayed at 37 °C in TKM buffer (25 mM Tris [pH 8.0], 100 mM KCl, 10 mM MgCl_2_, and 1 mM DTT). Reactions were initiated by the addition of 1× ATP regeneration mixture (75 μg/ml creatine kinase, 15 mM creatine phosphate, and 4 mM ATP). Samples were withdrawn at the indicated time points, quenched with 6× SDS-loading dye, separated by 15% SDS-PAGE, and detected by Coomassie brilliant blue staining. For heat denaturation experiments, either SmiA, GST-SwrA, or α-casein were incubated in TKM buffer at the indicated temperatures for 15 min. The reactions were then initiated by the addition of LonA_6_ (0.17 μM) and ATP regeneration mix. Samples were incubated at 37 °C for 10 min and quenched with 6× SDS-loading dye, and protein was detected as described.

### Fluorescence-based *in vitro* proteolysis assay

Degradation of GST-mNG-SwrA was monitored as a loss of fluorescence over time. 20 μl reactions prepared in TKM buffer containing the indicated concentrations of proteins were initiated by the addition of 1× ATP regeneration mix (see aforementioned one). Reactions were performed in triplicate at 37 ˚C and monitored by a BioTek plate reader (384-well format) with excitation and emission spectra of 490 and 520 nm, respectively. For assays containing 1.5 μM GST-mNG-SwrA, the excitation and emission spectra were 440 and 520 nm, respectively. When appropriate, the data were fit to a nonlinear least-squared fit of the Michaelis–Menten equation to obtain the *V*_max_ and *K*_*m*_. Reported fluorescent and kinetic values were the averages of (n = 3) ± 1 SD.

### *In vitro* pull-down assay

Reactions were prepared in 250 μl T(1) buffer (25 mM Tris [pH 8.0], 100 mM KCl, 10 mM MgCl_2_, 0.02% NP-40, 1 mM DTT, and 5 mM ATP) containing 0.5 μM SmiA, 0.5 μM GST-SwrA, or 80 nM LonA^K720Q^-His_6_ (monomeric). Each reaction series was incubated statically at 37 ˚C for 20 min. About 250 μl of 50% Glutathione-Sepharose resin slurry equilibrated with T(0) buffer (25 mM Tris [pH 8.0], 100 mM KCl, 10 mM MgCl_2_, and 0.02% NP-40) was added to each reaction series and incubated at room temperature with gentle agitation for 1 h. The resin was pelleted by centrifugation, and the supernatant was removed. The remaining pellets were washed three times with 250 μl T(1) buffer, followed by centrifugation and aspiration of the supernatant. The pellets were resuspended in T(1) buffer to a final volume of 250 μl, and 6× SDS-loading dye was added to both the pellet and supernatant fractions. Samples were then boiled for 10 min at 95 °C and subjected to Western blot analysis.

### Western blotting

Prepared samples were separated by 15% SDS-PAGE, and the proteins were electroblotted onto nitrocellulose membrane (GE Healthcare). The immunoblots were probed with anti-SmiA primary antibody (1:4000 dilution), anti-SwrA primary antibody (1:4000 dilution), anti-LonA primary antibody (1:10,000 dilution), and horseradish peroxidase–conjugated goat anti-rabbit immunoglobulin secondary antibody (1:10,000 dilution). The immunoblot was developed using the Peirce ECL Western blotting substrate kit (Thermo Fisher Scientific).

### Biolayer interferometry (BLI)

Anti-penta-His (HIS1K) biosensors (ForteBio) were hydrated by incubating in assay buffer (1× PBS, 1× kinetics buffer [ForteBio], 2% dialyzed fetal bovine serum, and 0.5 mM Tris(2-carboxyethyl)phosphine) for at least 10 min prior to beginning assay. The ligand His_6_-SmiA (300 nM) was loaded onto HIS1K biosensors for 200 s. Various concentrations of the analyte(s) GST-SwrA^WT^, GST-SwrA^Δ101–117^, or GST-SwrA^Δ1–100^ (50–400 nM) were used to measure the association kinetics for 100 s, after which the sensors were subsequently exposed to fresh assay buffer for 500 s to measure disassociation kinetics. The resulting association curve (0–100 s) and disassociation curve (0–300 s) were analyzed using the Octet RED96 instrument, and the data were processed using the ForteBio software. The binding data from the ligand sensors were normalized by subtracting a reference ligand sensor that was not exposed to GST-SwrA. The data were fit by a 1:1 binding model to obtain the *K*_*D*_. For LonA-binding experiments, the assay buffer was supplemented with 5 mM ATP, and 50 nM LonA-His_6_ was used as the ligand, and 200 to 800 nM SmiA was used as the analyte. The resulting association curve (0–500 s) and disassociation curve (0–500 s) were analyzed and processed as described. To determine the binding kinetics of unfolded SwrA, 600 nM His_6_-SmiA was used as the ligand, and the analyte GST-SwrA^WT^ (75–600 nM) was thermally denatured at 60 °C for 15 min prior to beginning the assay. The resulting association curve (0–500 s) and disassociation curve (0–500 s) were analyzed and processed as described. Analyzed data are presented in Table S1.

### Data analysis

All kinetic data were processed using GraphPad Prism software (GraphPad Software, Inc). For statistical significance tests, data were analyzed using ordinary one-way ANOVA tests. FIJI software (ImageJ) was utilized for densitometry analysis.

## Data availability

All data for this article are contained within the text or supporting information.

## Supporting information

This article contains supporting information.

## Conflict of interest

The authors declare that they have no conflicts of interest with the contents of this article.
