# Supplementary material for: SmiA is a hybrid priming/scaffolding adaptor for the LonA protease in Bacillus subtilis
Source: J Biol Chem. 2022 May 18;298(7):102045. doi: 10.1016/j.jbc.2022.102045 (PMC9204741; doi:10.1016/j.jbc.2022.102045)
Supplement: Supplemental Figures S1, S2 and Tables S1–S3 [file mmc1.pdf]

SmiA is a hybrid priming/scaffolding adaptor for the LonA protease in Bacillus subtilis

Stephen G. Olney, Peter Chien and Daniel B. Kearns

SUPPORTING INFORMATION

Fig S1

Fig S2

Table S1

Table S2

Table S3

Figure S1

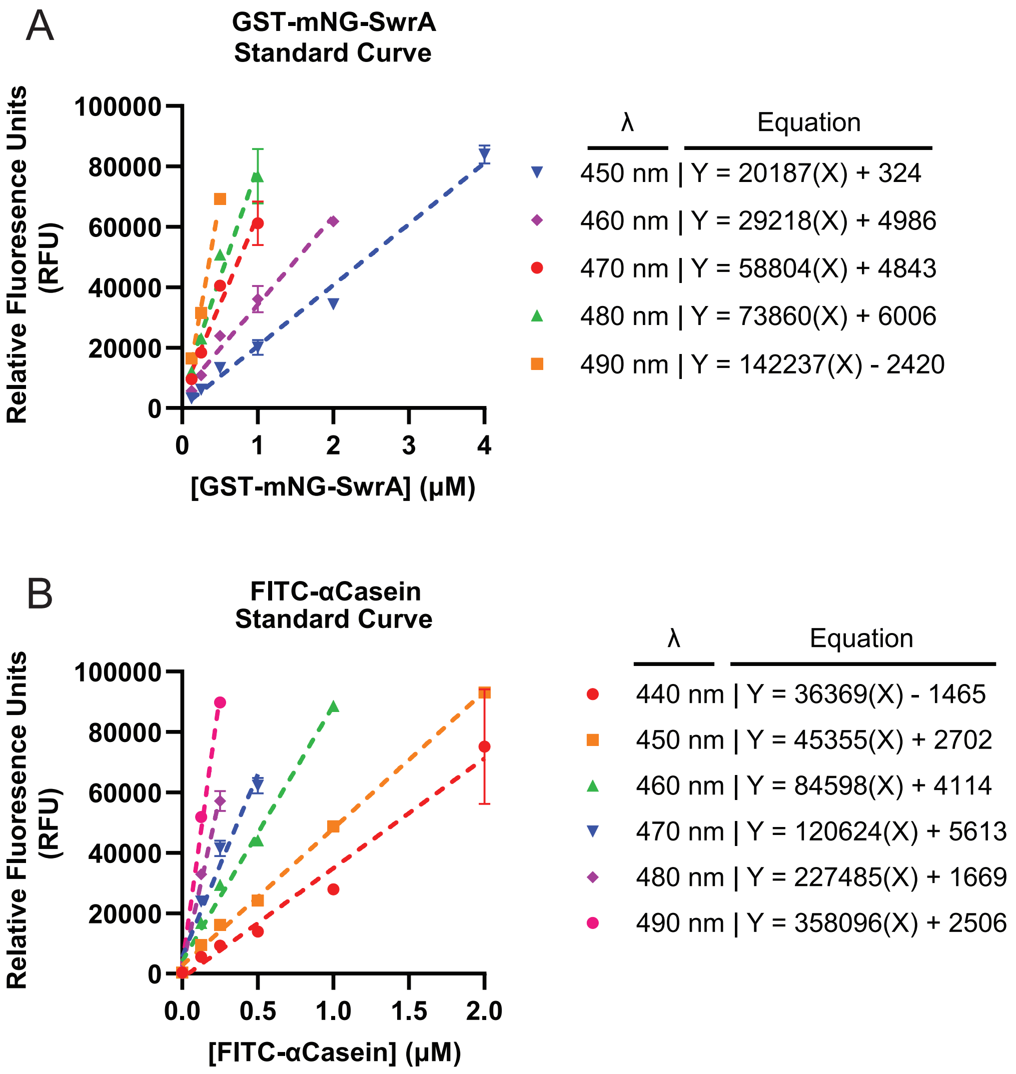

Figure S1. Standard curves for fluorescence to molecule conversion. A) Relative fluorescence units as a function of GST-mNG-SwrA concentration at a variety of excitation wavelengths. B) Relative fluorescence units as a function of FITC-α-casein concentration after degradation by LonA at a variety of excitation wavelengths. Degradation of α-casein was necessary to obtain accurate reading as FITC is known to quench fluorescence when covalently bound to a substrate.

Figure S2

```
                                                                                 WALKER A
BsLonA    1   --MAEELKRSIPLLPLRGLLVYPTMVLHLDVGRDKSVQALEQAMMHDHMIFLATQQDISI
EcLon     1   MNPERSERIEIPVLPLRDVVVYPHMVIPLFVGREKSIRCLEAAMDHDKKIMLVAQKEAST

BsLonA   59   DEPGEDEIFTVGTYTKIKQMLKLPNGTIRVLVEGLKRAHIVKYNEHEDYTSVDIQLIHED
EcLon    61   DEPGVNDLFTVGTVASILQMLKLPDGTVKVLVEGLQRARISALSDNGEHFSAKAEYLESP

BsLonA  119   DSKDTEDEALMRTLLDHFDQYIKISKKISAETYAAVTDIEEPGRMADIVASHLPLKLKDK
EcLon   121   TIDEREQEVLVRTAISQFEGYIKLNKKIPPEVLTSLNSIDDPARLADTIAAHMPLKLADK

BsLonA  179   QDILETADVKDRLNKVIDFINNEKEVLEIEKKIGQRVKRSMERTQKEYYLREQMKAIQKE
EcLon   181   QSVLEMSDVNERLEYLMAMMESEIDLLQVEKRIRNRVKKQMEKSQREYYLNEQMKAIQKE

BsLonA  239   LGDKEGKTGEVQTLTEKIEEAGMPDHVKETALKELNRYEKIPSSSAESSVIRNYIDWLVA
EcLon   241   LGEMDDAPDENEALKRKIDAAKMPKEAKEKAEAELQKLKMMSPMSAEATVVRGYIDWMVQ

                                                                WALKER A
BsLonA  299   LPWTDETDDKLDLKEAGRLLDEEHHGLEKVKERILEYLAVQKLTKSLKGPILCLAGPPGV
EcLon   301   VPWNARSKVKKDLRQAQEILDTDHYGLERVKDRILEYLAVQSRVNKIKGPILCLVGPPGV

BsLonA  359   GKTSLAKSIAKSLGRKFVRISLGGVRDESEIRGHRRTYVGAMPGRIIQGMKKAGKLNPVF
EcLon   361   GKTSLGQSIAKATGRKYVRMALGGVRDEAEIRGHRRTYIGSMPGKLIQKMAKVGVKNPLF

              WALKER B
BsLonA  419   LLDEIDKMSSDFRGDPSSAMLEVLDPEQNSSFSDHYIEETFDLSKVLFIATANNLATIPG
EcLon   421   LLDEIDKMSSDMRGDPASALLEVLDPEQNVAFSDHYLEVDYDLSDVMFVATSNS-MNIPA

BsLonA  479   PLRDRMEIINIAGYTEIEKLEIVKDHLLPKQIKEHGLKKSNLQLRDQAILDIIRYYTREA
EcLon   480   PLLDRMEVIRLSGYTEDEKLNIAKRHLLPKQIERNALKKGELTVDDSAIIGIIRYYTREA

BsLonA  539   GVRSLERQLAAICRKAAKAIVAE-ERKRITVTEKNLQDFIGKRIFRYGQAETEDQVGVVT
EcLon   540   GVRGLEREISKLCRKAVKQLLLDKSLKHIEINGDNLHDYLGVQRFDYGRADNENRVGQVT

BsLonA  598   GLAYTTVGGDTLSIEVSLSPGKGKLILTGKLGDVMRESAQAAFSYVRSKTEELGIEPDFH
EcLon   600   GLAWTEVGGDLLTIETACVPGKGKLTYTGSLGEVMQESIQAALTVVRARAEKLGINPDFY

                                 S677
BsLonA  658   EKYDIHIHVPEGAVPKDGPSAGITMATALVSALTGRAVSREVGMTGEITLRGRVLPIGGL
EcLon   660   EKRDIHVHVPEGATPKDGPSAGIAMCTALVSCLTGNPVRADVAMTGEITLRGQVLPIGGL

                K720
BsLonA  718   KEKALGAHRAGLTTIIAPKDNEKDIEDIPESVREGLTFILASHLDEVLEHALVGEKK---
EcLon   720   KEKLLAAHRGGIKTVLIPFENKRDLEEIPDNVIADLDIHPVKRIEEVLTLALQNEPSGMQ

BsLonA        -----
EcLon   780   VVTAK
```

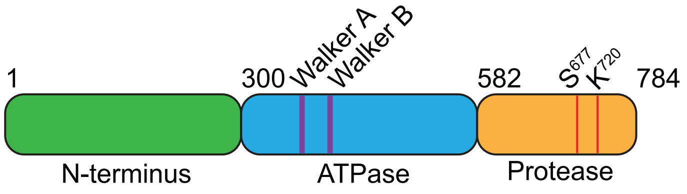

Figure S2. Lon protease family members are highly conserved. Multiple sequence alignment of B. subtilis LonA (BsLonA) and E. coli Lon (EcLon). Black highlight indicates identical residues, gray highlight indicates similar residues. Purple highlight indicates location of the Walker A and Walker B, ATP binding and hydrolysis sites. The protease active site residue K720 is labeled in red.

Table S1

Ligand	Analyte	K_D (M)	K_D error	k_{on} (M^{-1} s^{-1})	k_{on} error	k_{off} (s^{-1})	k_{off} error	Full X^2	Full R^2
His_6-SmiA	GST-$SwrA^{WT}$	6.09E-08	5.83E-09	1.53E+05	1.40E+04	9.30E-03	2.64E-04	12.1859	0.8199
His_6-SmiA	GST-$SwrA^{D101-117}$	4.32E-08	1.43E-09	9.68E+04	3.00E+03	4.18E-03	4.74E-05	6.6689	0.9632
His_6-SmiA	GST-$SwrA^{D1-100}$	N/A	N/A	N/A	N/A	N/A	N/A	N/A	N/A
His_6-SmiA	GST-$SwrA^{denat.}$	N/A	N/A	N/A	N/A	N/A	N/A	N/A	N/A
LonA-His_6	SmiA	N/A	N/A	N/A	N/A	N/A	N/A	N/A	N/A

Table S2

Plasmid	Genotype	Reference
pSS920	P_{T7}-*His*$_6$-*TEV-SmiA*	This study
pSM94	P_{T7}-*GST-SwrA*	Mukherjee *et al.,* 2015
pACB60	P_{T7}-*LonA-His*$_6$	Hughes *et al*., 2018
pSO31	P_{T7}-*LonA*^{K720Q}-*His*$_6$	This study
pSO31	P_{T7}-*GST-mNeonGreen-SwrA*	This study
pSO46	P_{T7}-*GST-mNeonGreen-SwrA*^{S107L}	This study
pSO47	P_{T7}-*GST-SwrA*^{S107L}	This study
pSO51	P_{T7}-*GST-mNeonGreen-SwrA*$^{\Delta 1\text{-}100}$	This study
pSO52	P_{T7}-*GST-mNeonGreen-SwrA*$^{\Delta 101\text{-}117}$	This study
pSO54	P_{T7}-*GST-SwrA*$^{\Delta 101\text{-}117}$	This study

Table S3

Construct	Primer	Sequence
pSS920	6497	CCTGTATTTTCAGGGCGCCATGGCCAGTGCCTTGAAGAGCATTG
	6498	CGTCGACGTAGGCCTTTGAATTCTTAATTGATTAAAAAGGGTTCCATAC
pSO31	4574	cctcctccatggcagaagaattaaaacgcagc
	4575	aggagggaattccttcatgcatggaattggtcgttc
pSO45	7546	CGTGGATCTCGTCGTGCATCTGTTATGGCTAGCCTCCCAGCG
	7547	cgcacaatacttgccctcttcatGAATTCGCCAGAACCAGCAGCGGAGCCAGCGGATCCCTTATAGAGTTCATCCATACCC
pSO46	7581	ggatttttcactaTTGaatgtcacgctgtgg
	7582	ccacagcgtgacattCAAtagtgaaaaatcc
pSO47	7581	ggatttttcactaTTGaatgtcacgctgtgg
	7582	ccacagcgtgacattCAAtagtgaaaaatcc
pSO51	7625	aggagCTCGAGttaaaggatttttcactatcgaatg
	7626	aggagCTCGAGGAATTCGCCAGAACCAGCAG
pSO52	7627	gatgtaaaccactatTAAaaggatttttcactatcg
	7628	cgatagtgaaaaatcctttTTAatagtggtttacatc
pSO54	7627	gatgtaaaccactatTAAaaggatttttcactatcg
	7628	cgatagtgaaaaatcctttTTAatagtggtttacatc
